# (Alkyl-ω-ol)triphenyltin(IV)-Loaded Mesoporous Silica as Biocompatible Potential Neuroprotectors: Evaluation of Inhibitory Activity Against Enzymes Associated with the Pathophysiology of Alzheimer’s Disease

**DOI:** 10.3390/nano15120914

**Published:** 2025-06-12

**Authors:** Kristina Milisavljević, Žiko Milanović, Jovana Matić, Marko Antonijević, Vladimir Simić, Miljan Milošević, Marijana Kosanić, Goran N. Kaluđerović

**Affiliations:** 1 Institute for Information Technologies, University of Kragujevac, Jovana Cvijića bb, 34000 Kragujevac, Serbia; kristina.milisavljevic@uni.kg.ac.rs (K.M.); jovana.todorovic@pmf.kg.ac.rs (J.M.); mantonijevic@uni.kg.ac.rs (M.A.); vsimic@kg.ac.rs (V.S.); miljan.m@kg.ac.rs (M.M.); 2 Department of Chemistry, Faculty of Science, University of Kragujevac, Radoja Domanovića 12, 34000 Kragujevac, Serbia; 3 Department of Biology, Faculty of Science, University of Kragujevac, Radoja Domanovića 12, 34000 Kragujevac, Serbia; marijana.kosanic@pmf.kg.ac.rs; 4 Faculty of Information Technology, Belgrade Metropolitan University, Tadeuša Koćuška 63, 11000 Belgrade, Serbia; 5 Department of Engineering and Natural Sciences, University of Applied Sciences Merseburg, Eberhard-Leibnitz-Straße 2, 06217 Merseburg, Germany

**Keywords:** Alzheimer’s disease, triphenyltin(IV) compounds, mesoporous silica SBA-15, molecular docking, molecular dynamics, computational brain model

## Abstract

Alzheimer’s disease (AD) is a progressive neurodegenerative disorder characterized by synaptic dysfunction and neuronal loss due to the accumulation of amyloid-β peptides and tau proteins. In the pursuit of novel neuroprotective strategies, organotin(IV) compounds have garnered attention due to their unique chemical and biological properties. This study evaluates the inhibitory potential of two triphenyltin(IV) derivatives—(3-propan-1-ol)triphenyltin(IV) (**Ph_3_SnL_1_**) and (4-butan-1-ol)triphenyltin(IV) (**Ph_3_SnL_2_**)—in both free form and immobilized into mesoporous silica SBA-15~Cl, targeting acetylcholinesterase (**AChE**), a key enzyme involved in AD pathophysiology. The **SBA-15~Cl|Ph_3_SnL_2_** nanostructures exhibited the most potent inhibitory activity against **AChE** (IC_50_ = 0.58 μM), significantly outperforming the standard drug galantamine. Molecular docking, molecular dynamics simulations, and MM/GBSA and MM/PBSA analyses confirmed the stability and selectivity of interactions with **AChE**, primarily driven by hydrophobic interactions. Compound transport was also simulated using a multi-scale 3D mouse brain model to evaluate brain tissue distribution and blood–brain barrier permeability. The results highlight the strong potential of SBA-15-loaded organotin(IV) compounds as biocompatible neuroprotective agents for novel treatments of neurodegenerative diseases.

## 1. Introduction

Alzheimer’s disease (AD) represents the most common form of neurodegenerative disorders. It is the primary cause of dementia in older adults, becoming a significant public health issue on a global scale [1,2]. The disease is characterized by the progressive deterioration of neurons, resulting in memory deficits, cognitive decline, behavioral changes, and reduced capacity to manage everyday tasks [3]. On a molecular level, Alzheimer’s disease involves the abnormal buildup of amyloid-beta (Aβ) aggregates outside neurons and the formation of neurofibrillary tangles composed of hyperphosphorylated tau protein within neuronal cells [4]. These pathological features trigger toxic pathways that ultimately impair synaptic function and lead to neuronal loss. Despite extensive investigation and various theories proposed to explain the underlying mechanisms—including amyloid-beta deposition [5], tau protein abnormalities [6], and disruption of cholinergic transmission [7]—an effective treatment capable of preventing or reversing disease progression remains elusive.

One of the primary therapeutic strategies in combating neurodegenerative diseases focuses on inhibiting enzymes involved in neurotransmitter regulation and oxidative stress [8,9,10]. Among these enzymes, acetylcholinesterase (**AChE**) plays a crucial role in the rapid degradation of acetylcholine in both the central and peripheral nervous systems [11]. Its main function is to regulate neurotransmission by hydrolyzing acetylcholine at the synapse. However, **AChE** is also implicated in the pathology of Alzheimer’s disease (AD) due to its involvement in amyloid plaque formation [11,12]. It has been shown to accelerate the aggregation of β-amyloid (Aβ) peptides into toxic fibrils, facilitated by interactions at its peripheral anionic site (PAS), which promotes Aβ oligomerization and plaque deposition. Inhibitors targeting PAS can effectively block AChE-mediated Aβ aggregation, while active site inhibitors primarily function by prolonging acetylcholine availability within the synapse [13,14]. Recent findings suggest that **AChE** also contributes to neuroinflammation and oxidative stress through non-cholinergic mechanisms, further exacerbating neuronal damage in AD [15,16]. Additionally, elevated **AChE** activity has been correlated with disease progression and severity, making it not only a therapeutic target for symptomatic relief, but also a potential modulator of disease pathology [15,17]. As a result, dual-binding inhibitors that interact with both the catalytic active site (CAS) and PAS are considered particularly promising, as they may simultaneously restore cholinergic function and inhibit amyloidogenesis [18,19,20]. The development of novel **AChE** inhibitors that combine favorable binding affinity, target selectivity, and the ability to modulate multiple pathogenic pathways thus remains a key focus in the search for more effective treatments for AD.

Organotin(IV) compounds represent a significant class of organometallic compounds with a wide range of applications in pharmacology. Their unique structure, based on tin atoms bonded to organic ligands, imparts high reactivity and selectivity toward various biological targets [20,21]. To enhance their stability, bioavailability, and efficacy, carriers are often used to enable controlled release and targeted delivery of active substances. Among them, mesoporous silica SBA-15 (Santa Barbara Amorphous-15) stands out due to its large specific surface area, tunable pore size, and chemical stability, making it suitable for the immobilization and transport of therapeutic compounds [22,23]. These carriers improve drug distribution and are particularly valuable in neurodegenerative disorder research due to their potential to overcome limited permeability through the blood–brain barrier [24].

**Figure 1:**
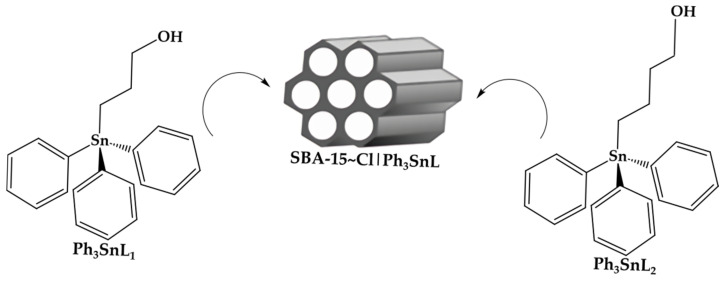
Structures of the investigated compounds: (3-propan-1-ol)triphenyltin(IV) (**Ph_3_SnL_1_**) and (4-butan-1-ol)triphenyltin(IV) (**Ph_3_SnL_2_**).

In the pharmacological context, organotin(IV) compounds have been investigated for their anticancer [25], antimicrobial [26], and antiviral properties [27], as well as for their potential to modulate enzymatic activities [28]. In this study, the structural parameters and inhibitory potential of specific organotin(IV) compounds, with and without the **SBA-15~Cl** carrier, were evaluated against key enzymes involved in the pathophysiology of Alzheimer’s disease: **AChE**, **COMT**, and **MAO-B** (Figure 1). These compounds were previously synthesized and structurally characterized [29,30,31,32,33]. Earlier research demonstrated the significant antitumor potential of organotin(IV) compounds in melanoma and ovarian carcinoma models, highlighting their therapeutic versatility and biological activity.

The objective of this study is to evaluate the potential of organotin(IV) compounds as neuroprotective agents through their inhibitory activity toward **AChE**, a key enzyme implicated in the progression of neurodegenerative diseases such as Alzheimer’s. This investigation is justified by the known anticancer properties, structural stability, and favorable electronic characteristics of organotin(IV) compounds, which also enable them to form stable interactions with biological macromolecules, particularly enzymes. To assess their therapeutic potential, we employed a finite element model of a mouse brain containing a blood vessel network to simulate the flow, permeability, and distribution of the selected compounds. This in silico model allowed us to investigate their ability to cross the blood–brain barrier and accumulate in brain tissue. Such insights are essential for determining bioavailability and enzyme-binding potential, which are essential prerequisites for the development of new therapeutic strategies targeting neurodegenerative conditions. 

## 2. Materials and Methods

### 2.1. Synthesis of Investigated Compounds

The synthesis of (3-propan-1-ol)triphenyltin(IV) (**Ph_3_SnL_1_**) and (4-butan-1-ol)triphenyltin(IV) (**Ph_3_SnL_2_**) was performed according to a previously reported method, where triphenyltin chloride (**Ph_3_SnCl**) reacts with respective Grignard reagent derived from Mg and 2-(3/4-halogenoalkoxy)tetrahydro-2*H*-pyrane, followed by deprotection of the hydroxyl group with PPTS and purification through column chromatography and recrystallization [29,30,31,32,33]. Immobilization was achieved by loading the compounds into the 3-(trimethoxysilyl)propyl chloride-functionalized SBA-15 (→**SBA-15~Cl**) described previously [29,30]. The mass content of organotin in the final hybrid materials was determined to be 4.35% for **SBA-15~Cl|Ph_3_SnL_1_** and 4.46% for **SBA-15~Cl|Ph_3_SnL_2_** [29,30,31,32,33]. The changes in the textural properties of SBA-15~Cl after the adsorption of organotin(IV) complexes have been confirmed in previous investigations. The initial pore diameter of SBA-15 is 4.97 nm, which decreased to 4.88 nm upon grafting (**SBA-15~Cl**); immobilization with **Ph_3_SnL_1_** yielded a pore size of 4.88 nm, while with **Ph_3_SnL_2_**, it decreased to 4.79 nm^2^. This reduction is accompanied by a notable decrease in the specific surface area (SBET), from 932 (SBA-15) and 535 m^2^/g (SBA-15~Cl) to 417 m^2^/g (**Ph_3_SnL_1_**) and 371 m^2^/g (**Ph_3_SnL_2_**), respectively, as well as a decline in total pore volume (Vm) from 0.94 cm^3^/g (SBA-15) and 0.58 cm^3^/g (**SBA-15~Cl**) to 0.49 cm^3^/g (**Ph_3_SnL_1_**) and 0.46 cm^3^/g (**Ph_3_SnL_2_**). These results indicate partial pore blockage due to the incorporation of the Ph3SnLn complexes into the mesoporous channels of **SBA-15~Cl**, confirming the successful functionalization of the material [29].

### 2.2. Ellman’s Method for AChE Inhibition Assay

The capacity of compounds to inhibit **AChE** served as the indicator of the neuroprotective activity of compounds. The rate of inhibition of **AChE** activity was determined spectrophotometrically using 96-well microtiter plates [34,35,36]. Acetylcholine iodide (**AChI**) was used as a substrate for the enzyme **AChE**, which degrades this compound into acetate and thiocholine. In the next step, 5,5′-dithiobis (2-nitrobenzoic acid) (DTNB) was transformed with thiocholine into a yellow-colored 5-thio-2-nitrobenzoate anion, and the change in absorbance was recorded on an ELISA plate reader (PKL PPC 142, Pokler Italia, Paramedical srl, Salerno, Italy) at 412 nm, 25 °C. 

The reaction mixture contained 140 μL of 0.1 mM sodium phosphate buffer (pH 8), 20 μL of DTNB (0.01 M; in 0.01 mM sodium phosphate buffer, pH 8), 20 μL of compounds (62.5, 31.25, 15.62, and 7.81 μg/mL), 20 μL of **AChE** (5 U/mL; in 0.02 M, pH 7.5 Tris buffer), and 10 μL of AChI (0.075 M; in 0.01 mM sodium phosphate buffer, pH 8). Galantamine was used as the positive control, while the negative control contained the same ingredients except for the compound (5% DMSO was used instead). A mixture of 5% DMSO and sodium phosphate buffer (0.01 mM; pH 8) was used as a blank. The rate of **AChE** inhibition (AChE(%)) was calculated using the following equation:

(1) $$AChE(\%)=\left[\frac{\left(E-S\right)}{E}\right]\times 100$$

where *E* is the enzyme activity without the compound, and *S* is the enzyme activity with the compound. The obtained values were compared with the commercial inhibitor of **AChE**, i.e., galantamine [34,35,36]. IC_50_ (the half-maximal inhibitory concentration) was used to present the neuroprotective activity of compounds. Lower IC_50_ values served as indicators of the activity of compounds. For **SBA-15~Cl**-supported systems, the mass content of the incorporated triphenyltin(IV) species (4.35% for **SBA-15~Cl|Ph_3_SnL_1_** and 4.46% for **SBA-15~Cl|Ph_3_SnL_2_**) was taken into account when calculating the effective concentration of the active component, ensuring accurate determination of IC_50_ values [29,30,31,32,33].

### 2.3. Computational Methods

#### 2.3.1. DFT Method and Diffusion Coefficient Calculation

All computational calculations were conducted using the Gaussian09 software package [37], with GaussView 6.0.16 utilized for molecular visualization and structural analysis [38]. Geometry optimizations of the ligand structures were carried out employing density functional theory (DFT), specifically utilizing the B3LYP-D3BJ functional in combination with the 6-311++G(d,p) basis set in order to obtain their most stable conformations prior to docking. This combination incorporates both polarization and diffuse functions, which significantly enhance the accuracy of the calculated molecular properties [39,40].

The B3LYP-D3BJ method was selected due to its proven accuracy in reproducing experimental geometric parameters, as demonstrated by numerous previous studies [41,42,43,44]. This function offers an optimal balance between computational efficiency and precision, making it particularly suitable for the study of complex molecular systems. The inclusion of Grimme’s D3BJ dispersion correction effectively accounts for long-range van der Waals interactions, which are essential for accurately modeling non-covalent interactions present in the investigated compounds [45]. Furthermore, the use of the 6-311++G(d,p) basis set ensures a thorough description of both valence and diffuse electron distributions, enabling precise characterization of complex molecular electronic structures.

After obtaining the optimized geometries in the gas phase, the structures were reoptimized in an aqueous environment to better represent physiological conditions and calculate the diffusion coefficients. To estimate the diffusion coefficients (D) of the compounds, the Stokes–Einstein equation was employed [46,47,48]:

(2) $$D=\frac{k_{B}T}{6\pi \eta \alpha }$$

where α is the hydrodynamic radius of each compound, k_B_ is the Boltzmann constant (1.380 × 10^−23^ J/K), and η represents the viscosity of water under physiological conditions (8.905 × 10^−4^ Pa·s) [49]. The reoptimized structures in water yielded the hydrodynamic radii, important for calculating the diffusion coefficients. These calculations were fundamental in determining the parameters required to simulate drug flow through a mouse brain model, offering valuable insights into the compounds’ mobility and distribution under physiological conditions. This integrated approach, combining DFT optimization with diffusion coefficient estimation, enables a thorough understanding of the compounds’ dynamic behavior within the brain environment. 

#### 2.3.2. Molecular Docking Approach for Enzyme Inhibition Analysis

Molecular docking and molecular dynamics simulations were carried out to investigate the tested compounds’ interactions with the **AChE** receptor. To evaluate the binding affinity and potential inhibitory effects of these compounds on the targeted proteins, AutoDock 4.2 software [50] was used in combination with the Lamarckian genetic algorithm [51]. The Lamarckian genetic algorithm parameters were set to a maximum of 250,000 energy evaluations, 27,000 generations, and mutation and crossover rates of 0.02 and 0.8, respectively. AutoDockTools was utilized to incorporate Kollman partial charges and polar hydrogens. Protein structures were kept rigid during the simulations, while the flexibility of the ligands/complexes was considered. The molecular docking studies involved a systematic workflow, including protein identification and preparation, ligand preparation, and network formation. The structures of the compounds were optimized according to these protocols. The 3D X-ray crystallographic structure of **AChE** (PDB code 4EY7, accessed on 19 December 2024) [52] was retrieved from the RCSB Protein Data Bank. Before the docking studies, co-crystallized water molecules and ligands were removed from the binding sites using Discovery Studio 4.0 (BIOVIA, Dassault Systèmes, San Diego, CA, USA) to ensure a clean interaction environment. The search space for **AChE** was defined as a grid frame size of 50 × 50 × 50 Å, corresponding to the XYZ dimensions of −14.108 × −43.833 × 27.670, with a grid spacing of 0.375 Å. The grid was centered on the active site of **AChE**, using the donepezil inhibitor already present in the crystal structure as a reference point to accurately target the ligand-binding region. Other docking parameters followed standard protocols as outlined in our previous research [53,54,55,56,57].

#### 2.3.3. Computational Evaluation of Complex Stability via Molecular Dynamics Simulation

All molecular dynamics (MD) simulations have been performed utilizing the AMBER22 software package [58,59,60]. Protein–ligand complexes were constructed Via the tleap module, employing the ff19SB force field for the protein and the Generalized Amber Force Field 2 (GAFF2) for the ligands [60]. Metal ions were parameterized with the MCPB.py tool to guarantee a precise depiction of metal–ligand interactions [61]. The initial protein–ligand complexes were derived from molecular docking simulations. All systems were solubilized in a truncated octahedral box of TIP3P water, ensuring a minimum distance of 12 Å between the solute and the simulation box boundary. Counterions were introduced to equilibrate the system. Energy minimization was executed in two phases: an initial minimization involving a harmonic restraint of 500 kcal/mol/Å² on the solute, while only the solvent and ions were allowed to relax after over 5000 steps, succeeded by a comprehensive minimization in which all restraints were lifted, and the entire system underwent an additional 10,000 steps of minimization. The reduced structures were subsequently heated incrementally from 0 K to 300 K over 100 ps utilizing a Langevin thermostat with a collision frequency of 2 ps^−1^, while the solute atoms were constrained with a force constant of 10 kcal/mol/Å^2^ [62]. The subsequent stage involved 1 ns of equilibration at constant pressure (1 atm) and temperature (300 K), during which the positional restrictions were progressively decreased. Production molecular dynamics simulations were conducted with the CUDA implementation of the pmemd engine in AMBER22, facilitating effective GPU acceleration. A total of 100 nanoseconds of molecular dynamics simulations were performed under the NPT ensemble (constant particle number, pressure, and temperature). The SHAKE algorithm was utilized to restrict bonds involving hydrogen atoms, enabling a 2 fs time step [63]. Long-range electrostatic interactions were addressed Via the Particle Mesh Ewald (PME) approach, employing a 10 Å threshold for nonbonded interactions [64]. After the molecular dynamics simulations, the molecular mechanics Poisson–Boltzmann surface area (MM/PBSA) and molecular mechanics Generalized Born surface area (MM/GBSA) methods were utilized to calculate the binding free energies of the **AChE-Ph_3_SnL_1_** and **AChE-Ph_3_SnL_2_** ligands in their corresponding complexes. The computations were conducted using the MMPBSA.py module in AmberTools23. The overall binding free energy was analyzed by decomposing it into molecular mechanics energy components, which encompass van der Waals and electrostatic interactions, in addition to solvation-free energy contributions calculated Via the Generalized Born (GB)- and Poisson–Boltzmann (PB)-implicit solvent models [65].

### 2.4. Computational Modeling of Compound Transport

The initial modeling phase begins with extracting DICOM images from a micro-CT scan of a mouse brain. To prepare the sample, blood was flushed from the vasculature using ex vivo transcardial perfusion—first with a heparin sodium salt solution in 0.9% NaCl, followed by the injection of the contrast agent Microfil. After perfusion, the Microfil was allowed to solidify for several hours. The brain was then preserved in 4% paraformaldehyde. Micro-computed tomography (micro-CT) was performed by the Preclinical Imaging Core at the Houston Methodist Research Institute to visualize the vascular and tissue structure. The resulting DICOM images were processed using a combination of software tools, including Mimics Research Medical 20.0 and Geomagic Studio, to reconstruct a 3D model and generate an STL file of the brain tissue with its vasculature.

Compound transport was simulated using smeared modeling concepts and Kojic Transport Models, where the main idea is to consider that the whole continuum space is divided into two domains—capillary and tissue [66]. The elementary volumes occupied by those domains within the finite element at the considered (integration) points are r_V_dV for the capillary and (1 − r_V_)dV for the tissue domain. Four physical fields, mutually dependent, are present within the finite element: pressure and concentration in capillaries, and pressure and concentration within the tissue. The continuum domains can consist of extracellular space, cells, and organelles that are independent domains, with their own finite element mesh of continuum elements. Capillaries and lymph vessels are represented using one-dimensional elements immersed in extracellular space, where those domains have their own 1D finite elements with coordinate axes along the elements. 

Regarding the software architecture, PAK is an in-house-developed FEM software (https://github.com/BogdanM1/PAK-KTM, accessed 2 on April 2025), originally developed at the University of Kragujevac and BioIRC (Bioengineering Research and Development Center, Kragujevac, Serbia), and served as the computational engine for solving the governing equations of mass transport. Previous works display the application of the PAK in numerous complex biological systems [67,68,69,70].

For modeling the diffusion processes within the model, we have used Fick’s law formulations. The transport phenomena in both tissue and vascular domains were discretized using the standard Galerkin weighting method [71], converting the governing equations into finite element balance equations. The software’s architecture allowed for the efficient modeling and solution of the complex and coupled 1D-3D mouse brain system, where the model is composed of 1D finite elements—used for modeling the vascular network consisting of both capillaries and veins and fictitious elements to connect fluid, as well as 3D finite elements—to model the surrounding tissue space [72]. 

The second phase starts with importing the .stl file into our in-house software CAD. For geometric modeling and result interpretation, the integrated CAD Field and Solid software (https://github.com/miljanmilos/CAD-Solid-Field, accessed on 5 April 2025) [73]—developed in-house by the University of Kragujevac and BioIRC—serves as the pre-processing and post-processing tool. This software enables efficient mesh generation, boundary condition application, and advanced post-processing functionalities, including 3D visualization, animation, and quantitative analysis of simulation results. Its modularity and integration with the PAK solver significantly enhance the overall workflow for high-fidelity computational modeling.

## 3. Results and Discussion

### 3.1. Affinity to Acetylcholinesterase (AChE)

Although the exact cause of neurodegenerative diseases, particularly Alzheimer’s disease, remains unclear, it is known that these conditions are associated with reduced levels of acetylcholine or dopamine [74]. The enzyme **AChE** hydrolyzes acetylcholine, thereby terminating synaptic transmission. As a result, **AChE** inhibitors are considered the most effective treatment option for these disorders, as they help restore acetylcholine levels in cholinergic synapses by reducing the enzyme’s activity [15]. Since synthetic **AChE** inhibitors are costly and often cause various side effects, increasing attention is being directed toward discovering other alternatives.

**Figure 2:**
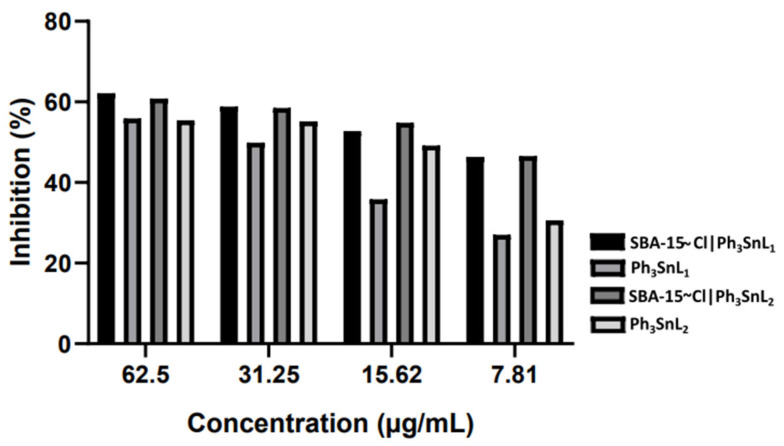
Inhibitory activity of free and SBA–15–immobilized triphenyltin(IV) compounds against acetylcholinesterase (**AChE**) at different concentrations. Results are expressed as a percentage of inhibition (%).

The neuroprotective activity of organotin(IV) compounds has been a subject of increasing interest over the past two decades, particularly considering their diverse biological effects and potential therapeutic applications [75]. For this reason, selected organotin(IV) compounds were evaluated for their ability to inhibit **AChE**, and the obtained ***apparent*** and ***corrected*** IC_50_ values are presented in Table 1. The corresponding inhibition profiles at different concentrations are illustrated in Figure 2. The apparent IC_50_ values reflect the concentration of the total functionalized material (i.e., organotin complex bound to the SBA-15 support) required to inhibit 50% of enzyme activity, without adjusting for the actual content of the active compound. In contrast, the corrected IC_50_ values were calculated by normalizing the inhibitory concentration to the amount of the active organotin(IV) complex, based on the Sn content determined by elemental analysis. This correction enables a more accurate comparison of the true inhibitory potency of the active compounds, independent of the carrier matrix. This study marks the first time these compounds have been tested for neuroprotective activity. The results reveal that all tested complexes exhibited **AChE** inhibition, with significant differences in potency. 

Among the compounds, **SBA-15~Cl|Ph_3_SnL_2_** demonstrated the highest inhibitory activity, with an IC_50_ of 0.58 µM, outperforming the positive control, galantamine (IC_50_ = 15.00 µM), by nearly 26-fold. Similarly, **SBA-15~Cl|Ph_3_SnL_1_** (IC_50_ = 1.07 µM) exhibited stronger inhibition than galantamine. These corrected IC_50_ values, calculated based on the actual content of organotin(IV) complexes in the mesoporous support, reveal the true potency of the active compounds and allow a more accurate comparison with standard inhibitors. Notably, the apparent IC_50_ values for these two materials, based on the total mass of the functionalized hybrid, were 13.44 µM and 23.93 µM for **SBA-15~Cl|Ph_3_SnL_2_** and **SBA-15~Cl|Ph_3_SnL_1_**, respectively, suggesting that these values reflect the activity of the overall system but may underestimate the intrinsic inhibitory capacity of the embedded organotin(IV) species.

In contrast, the free ligands, **Ph_3_SnL_1_** and **Ph_3_SnL_2_**, displayed significantly lower activity, with IC_50_ values of 105.81 µM and 88.00 µM, respectively. For comparison, the IC_50_ values for the inhibition of **AChE** by some other reported compounds are as follows: trigonelline (233 ± 0.12 µM) [76], rivastigmine (501 ± 3.08 µM) [76], different salicylanilide (thio)carbamates (ranging from 38.9 ± 1.1 µM to 89.7 ± 4.7 µM) [77], quercetin (54.5 µM), myricetin (43.2 µM), and carbamate derivatives (ranging from 29.9 to 105.4 nM) [78]. These data demonstrate that **SBA-15~Cl|Ph3SnL_1_** and **SBA-15~Cl|Ph3SnL_2_** exhibit superior inhibitory activity compared to many previously studied compounds, including the standard inhibitor galantamine.

These findings highlight the important role of the **SBA-15~Cl** carrier in enhancing the bioactivity of organotin(IV) complexes, as the **SBA-15~Cl**-supported compounds are significantly more potent than their free analogs. This suggests that the carrier system improves the availability or interaction of these complexes with the enzyme, leading to superior inhibitory activity. This enhancement is likely a result of the improved dispersion, physicochemical stability, and spatial orientation of the active complexes within the mesoporous structure, which increases their accessibility to the enzyme and prolongs interaction time. The incorporation of organotin(IV) compounds into the **SBA-15~Cl** framework significantly increases their inhibitory activity, aligning to improve efficacy through carrier-assisted delivery. Given that previous studies have demonstrated how **SBA-15~Cl** mesoporous matrices boosted the anticancer efficacy of these organotin(IV) compounds, such an outcome in **AChE** inhibition is to be expected, thus confirming that **SBA-15~Cl** also acts as a powerful enhancer of their neurobiological activity. Future studies should investigate their mechanism of action and potential therapeutic applications in neurodegenerative diseases.

### 3.2. Molecular Docking and Intermolecular Interactions with AChE

The molecular docking study provided valuable insights into the binding interactions of **Ph_3_SnL_1_** and **Ph_3_SnL_2_** with **AChE**, a key enzyme involved in the pathophysiology of Alzheimer’s disease. The results were compared with reference inhibitors galantamine, tolcapone, and safinamide to evaluate the relative efficacy of the synthesized compounds (Table 2). The **SBA-15~Cl** nanostructures (**SBA-15~Cl|Ph_3_SnL_1_** and **SBA-15~Cl|Ph_3_SnL_2_**) were not included in the molecular docking study due to the structural complexity and size of the hybrid materials, which exceed the limitations of standard docking protocols. These systems involve a solid support framework that cannot be accurately represented in conventional molecular docking simulations, which are optimized for discrete, soluble molecules. Therefore, only the free organotin(IV) compounds (**Ph_3_SnL_1_** and **Ph_3_SnL_2_**) were analyzed to assess their direct interactions with the enzyme active sites. 

For **AChE**, **Ph_3_SnL_2_** displayed stronger binding interactions than galantamine, suggesting a more stable and effective interaction with the enzyme’s active site. This enhanced binding is primarily attributed to favorable van der Waals and hydrogen bonding interactions, likely contributing to its higher inhibitory potential (Table 2). The predicted inhibition constants (K_i_) support this observation, as both **Ph_3_SnL_2_** and **Ph_3_SnL_1_** exhibited lower Ki values than galantamine, indicating stronger enzyme–ligand binding affinities. When compared with donepezil, a clinically approved FDA drug for Alzheimer’s disease, **Ph_3_SnL_2_** exhibited a slightly less favorable overall binding energy and a somewhat higher inhibition constant, yet demonstrated a comparable predicted binding affinity, suggesting competitive inhibitory potential in silico. **Ph_3_SnL_1_** also demonstrated a comparable interaction profile, positioning it within the effective range of known **AChE** inhibitors. Notably, these computational findings are in good agreement with experimental IC_50_ values, which also indicate that **Ph_3_SnL_2_** exhibits superior inhibitory activity compared to galantamine (Table 1). **Ph_3_SnL_1_** also showed competitive binding compared to the reference inhibitor, further indicating its potential as an **AChE** inhibitor.

**Figure 3:**
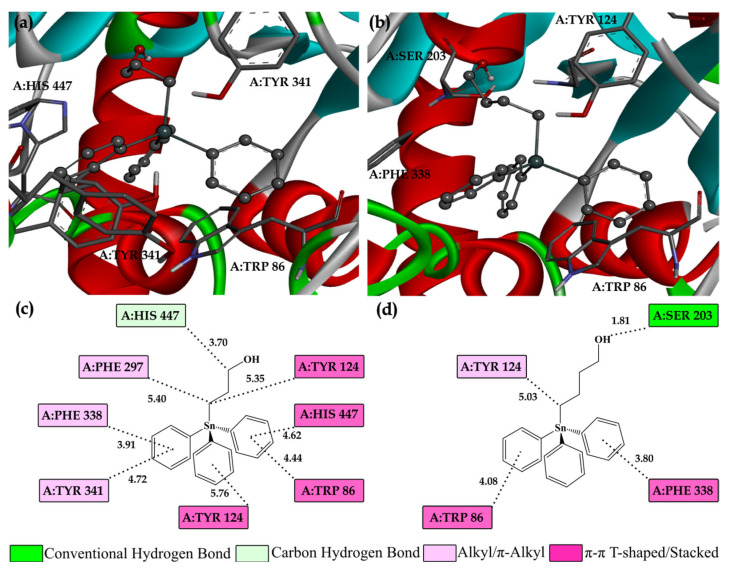
3D (**a**,**b**) and 2D (**c**,**d**) representations of the investigated compounds, **Ph_3_SnL_1_** and **Ph_3_SnL_2_**, within the active site of the **AChE** enzyme are presented. The red α-helices correspond to the helical regions of the **AChE** enzyme, which play a crucial role in maintaining the protein’s structural integrity and overall stability. The green loops and β-turns represent flexible regions of the enzyme, essential for facilitating conformational changes required for effective ligand binding. Numerical values, expressed in angstroms (Å), denote the interatomic distances, while different colors in the representations correspond to various types of intermolecular interactions, as detailed in the accompanying legend.

The analysis of intermolecular interactions between the enzymes and the investigated compounds represents a key step in understanding the mechanisms of inhibition, as it enables the identification of specific bonds that contribute to the stability of the complex and the overall binding affinity. These interactions directly influence the efficacy of the inhibitors and their selectivity toward the target enzyme. Using molecular docking, the energetically most favorable conformations of the ligands within the enzyme active sites were identified, and their characteristic interactions with amino acid residues are presented in Figure 3.

In the case of **AChE,** conventional hydrogen bonds play a critical role in stabilizing protein–ligand complexes by enabling direct interactions between the polar functional groups of the ligand and key amino acid residues within the enzyme’s active site (Figure 3). For instance, in the **Ph_3_SnL_2_–AChE** complex, the hydrogen atom from the hydroxyl group forms a conventional hydrogen bond with the residue A:SER203. The short interatomic distance, measuring less than 2.0 Å, reflects the strength and specificity of this interaction and underscores its contribution to the overall stability of the complex. Similarly, **Ph_3_SnL_1_** engages in a carbon–hydrogen bond with A:HIS447, representing another relevant type of hydrogen bonding that adds to the stabilization of the enzyme–ligand complex.

Hydrophobic interactions between the nonpolar regions of the ligand and the hydrophobic pockets in the active site further stabilize the complex. Alkyl and π-alkyl interactions play a critical role in anchoring the ligand within the enzyme’s active site. For instance, **Ph_3_SnL_1_** forms alkyl and π-alkyl interactions with key residues such as A:PHE297, A:PHE338, and A:TYR341, while **Ph_3_SnL_2_** engages in similar interactions with A:TYR124. These interactions enhance the stability of the complex by promoting specificity and increasing binding affinity (Figure 2). Moreover, both **Ph_3_SnL_1_** and **Ph_3_SnL_2_** participate in π-π interactions, which include T-shaped and stacked configurations. The aromatic rings of the ligands form strong π-π interactions with residues such as A:TRP86, A:PHE338, A:TYR124, and A:HIS447, thereby increasing the binding affinity and reinforcing the ligand’s positioning within the active site. Hydrophobic interactions, particularly those involving aromatic rings, are essential for stabilizing the ligand within the enzyme’s active site. These interactions highlight the importance of strategically positioning aromatic rings when designing potential inhibitors, as they significantly improve the stability of the protein–ligand complex.

### 3.3. Molecular Dynamics Simulations

#### 3.3.1. Structural and Dynamic Stability of AChE–Ligand Complexes

The molecular dynamics (MD) simulations of **AChE-Ph_3_SnL_1_** and **AChE-Ph_3_SnL_2_** systems in conjunction with the target protein yielded essential insights into their structural stability, conformational flexibility, and binding properties. An in-depth analysis of Root Mean Square Deviation (RMSD), Radius of Gyration (Rg), Root Mean Square Fluctuation (RMSF), and Number of Hydrogen Bonds (nHB) elucidates significant distinctions between the two ligands and their prospective effects on binding affinity and protein stability. 

**Figure 4:**
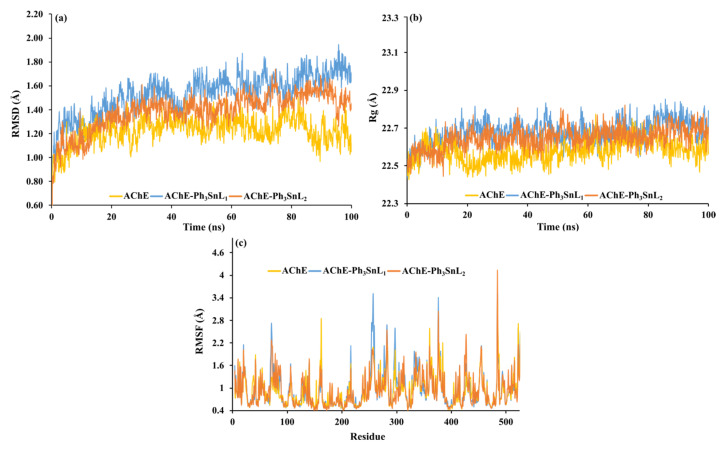
Structural stability and compactness analysis of **AChE–Ligand** complexes: (**a**) RMSD, (**b**) Radius of Gyration, and (**c**) RMSF variations over a 100 ns MD simulation.

RMSD analysis, a key metric for assessing global structural stability, revealed a well-defined equilibration phase within the initial 10–20 ns of the simulations, followed by trajectory stabilization across all systems, indicating successful convergence (Figure 4a). A comparative analysis of average RMSD values over the 100 ns simulation period demonstrated that the unbound **AChE** exhibited the lowest deviation (1.23 ± 0.12 Å), consistent with its inherent structural stability in the absence of ligands. In contrast, the presence of ligands **Ph_3_SnL_1_** and **Ph_3_SnL_2_** led to increased RMSD values, measured at (1.53 ± 0.18 Å) and (1.39 ± 0.16 Å), respectively. These findings suggest that ligand binding induces additional structural adjustments within the protein, with a more pronounced effect observed in the **AChE-Ph_3_SnL_1_** complex.

The elevated RMSD observed in the **AChE-Ph_3_SnL_1_** system points to greater conformational flexibility, potentially reflecting a more dynamic binding mode or less rigid interactions within the binding pocket. Conversely, the lower RMSD associated with the **AChE-Ph_3_SnL_2_** complex may indicate a more stable and tightly bound interaction with the protein, resulting in less structural perturbation. This behavior implies that **AChE-Ph_3_SnL_2_** may engage in more favorable or stabilizing contacts within the active site, which could have a positive influence on its inhibitory efficacy through enhanced complex stability.

The Rg was evaluated to monitor changes in the overall compactness and folding behavior of the **AChE** structure upon ligand binding. Rg values remained relatively stable throughout the 100 ns simulation for all systems, with minor oscillations confined to a narrow range (~22.4–22.8 Å), indicating the absence of significant unfolding events or global structural disruption (Figure 4b). The average Rg values were calculated as follows: 22.58 ± 0.06 Å for unbound **AChE**, 22.68 ± 0.06 Å for the **AChE-Ph_3_SnL_1_** complex, and 22.65 ± 0.06 Å for the **AChE-Ph_3_SnL_2_** complex. The slight increase in Rg observed upon ligand binding suggests a modest expansion of the protein structure, likely due to local conformational rearrangements or ligand-induced allosteric effects. Importantly, this increase is minimal and does not imply a loss in structural integrity. These findings support the notion that both ligands interact with **AChE** without compromising its global architecture. The maintenance of a consistent Rg profile further confirms that the complexes remain structurally compact and well folded throughout the simulation, reinforcing the overall stability of the ligand-bound systems. 

The analysis of the RMSF profiles (Figure 4c) provides a detailed overview of local fluctuations in individual amino acid residues throughout the simulation, offering insights into the flexibility of different regions of the protein structure. In the ligand-bound systems with **Ph_3_SnL_1_** and **Ph_3_SnL_2_**, increased fluctuations were primarily observed in loop regions and surface-exposed residues, consistent with the expected dynamic behavior of these structurally less stable segments.

The RMSF distributions display a similar pattern for both complexes, with the highest fluctuations localized near the active site, involving residues that are likely functionally significant for ligand binding. In contrast, the central regions of the protein, which form the structural core, exhibit markedly lower RMSF values (<1.5 Å), confirming their inherent rigidity and structural stability. Certain loop regions, however, show fluctuations exceeding 3.0 Å, highlighting zones of pronounced local dynamics.

Average RMSF values further support these observations: 1.05 Å for free **AChE**, 0.99 Å for the **AChE-Ph_3_SnL_1_** complex, and 0.94 Å for **AChE-Ph_3_SnL_2_**. The reduced average flexibility in the ligand-bound forms suggests a general stabilizing effect of ligand binding on the protein structure. Notably, the **AChE-Ph_3_SnL_1_** complex exhibits slightly more pronounced fluctuations at specific residues compared to **AChE-Ph_3_SnL_2_**, which may correspond to the higher RMSD values previously observed for this system and point toward a more dynamic binding mode. These localized differences in flexibility may influence the accommodation and stabilization of the ligand within the binding pocket, particularly given that the fluctuating regions often overlap with residues directly involved in ligand–protein interactions.

**Figure 5:**
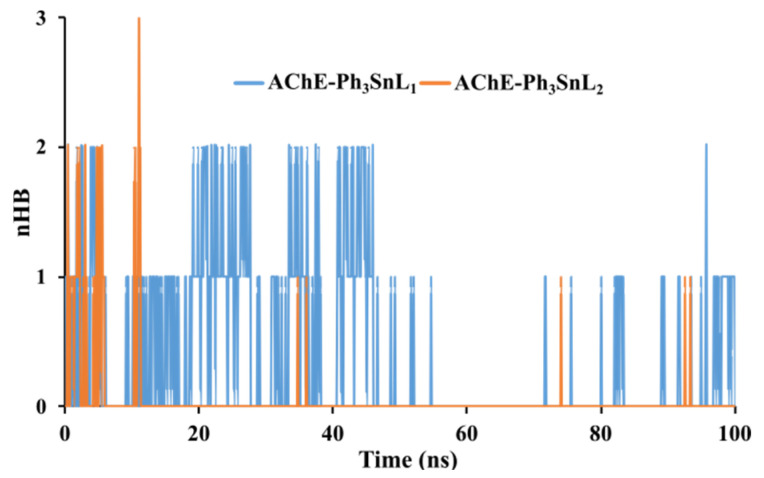
Hydrogen bonding analysis of **AChE–Ligand** complexes: Temporal evolution over 100 ns MD simulation.

Hydrogen bonding plays a critical role in determining the stability and specificity of ligand–protein interactions. Analysis of the number of hydrogen bonds (nHB) formed between the ligands and **AChE** throughout the 100 ns simulation revealed notable differences between the two systems. On average, the **AChE-Ph_3_SnL_1_** complex formed 0.37 hydrogen bonds, whereas **AChE-Ph_3_SnL_2_** formed only 0.06, indicating that **Ph_3_SnL_1_** engages more frequently in polar interactions with the protein (Figure 5).

Despite the relatively low absolute number of hydrogen bonds in both systems, the higher frequency observed for **Ph_3_SnL_1_** suggests a more favorable orientation or greater compatibility of this ligand within the binding site, at least concerning polar contacts. Nevertheless, the transient and dynamic nature of hydrogen bonding is evident, as fluctuations in nHB values were observed over time, reflecting the inherent flexibility of both the ligand and the binding pocket.

It is important to note that hydrogen bonds alone may not fully account for the overall stability of the complexes. The relatively low nHB values suggest that additional non-covalent interactions, such as hydrophobic contacts, van der Waals forces, or π-π stacking, may significantly contribute to ligand stabilization. This is particularly relevant for systems like **AChE-Ph_3_SnL_2_**, where hydrogen bonding is minimal, yet the complex remains structurally stable, as indicated by its lower RMSD and Rg values.

Moreover, a decreasing trend in the number of hydrogen bonds over the simulation timeframe, especially in the **AChE-Ph_3_SnL_1_** complex, mirrors the trends observed in RMSD and Rg analyses, potentially indicating conformational rearrangements within the binding site. These structural adaptations may alter the availability or orientation of hydrogen bond donors and acceptors, further supporting the dynamic character of ligand binding.

#### 3.3.2. MM/GBSA and MM/PBSA Binding Free Energy Estimates of AChE–Ligand Complexes

Binding free energy calculations provide critical insights into the thermodynamic favorability and strength of ligand–protein interactions. In this study, both MM/GBSA and MM/PBSA methods were employed to estimate the binding affinities of **Ph_3_SnL_1_** and **Ph_3_SnL_2_** toward **AChE**, revealing a pronounced discrepancy between the two approaches. As shown in Table 3, the MM/GBSA method predicted strongly favorable binding free energies for both complexes (−29.53 kcal/mol for **AChE-Ph_3_SnL_1_** and −32.55 kcal/mol for **AChE-Ph_3_SnL_2_**), while the MM/PBSA calculations yielded significantly less favorable values, with positive binding energy for **AChE-Ph_3_SnL_1_** (7.82 kcal/mol) and only marginally favorable binding for **AChE-Ph_3_SnL_2_** (1.84 kcal/mol).

However, according to the literature as well as our previous experiences, the presence of a metal center in the ligand can substantially influence the MM/GBSA and MM/PBSA energy calculations owing to its impacts on electrostatics, solvation energy, and ligand rigidness. Metal ions frequently demonstrate significant electrostatic interactions and necessitate explicit solvation effects for precise modeling, resulting in considerable discrepancies between the Generalized Born (GB) and Poisson–Boltzmann (PB) solvation models. The PB solvation model disproportionately penalizes metal-containing complexes due to its explicit consideration of polar solvation, frequently resulting in elevated (less favorable) binding free energy estimations relative to MM/GBSA. This mismatch occurs because PB models consider the significant desolvation penalty associated with transferring metal-coordinated ligands from aqueous environments to the binding pocket, while GB models offer a more approximate and often less punitive treatment of solvation effects. Moreover, Sn-containing ligands frequently exhibit reduced flexibility owing to the stiffness conferred by coordination interactions, which might fix the ligand inside the binding site and diminish MM/GBSA energy estimations. Nonetheless, if considerable electrostatic desolvation effects are detected, MM/PBSA simulations may exaggerate the instability of these complexes. Previous investigations have revealed analogous disparities between MM/GBSA and MM/PBSA for metal-containing complexes, highlighting the limits of implicit solvation models in precisely characterizing metallodrug interactions [79,80,81,82]. 

### 3.4. Modeling the Distribution of Compound Concentration in Brain Tissue

Presented mouse brain tissue geometry and vascular network are generated at the R&D Center for Bioengineering (BIOIRC) and the Institute for Information Technologies Kragujevac, using the imaging data obtained from animal studies conducted by a protocol approved by the Institutional Animal Care and Use Committee (IACUC) at the Laboratory of the Singleton Department of Pediatric Radiology, Texas Children’s Hospital. Comprehensive details regarding the study protocol and imaging procedures are provided in [73]. 

**Figure 6:**
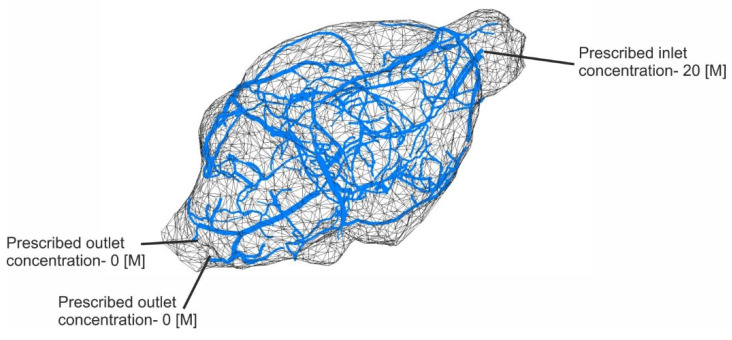
Finite element model of mouse brain; representation of large blood vessels (denoted blue in the figure) and the surrounding tissue surface (wireframe), with prescribed boundary conditions.

The finite element geometry of the mouse brain model is presented in Figure 6. The computational model consists of three parts: one-dimensional finite elements for larger vessels (6241 elements), three-dimensional composite smeared finite elements (173,876 elements) representing the surrounding tissue, and connectivity elements (874 elements) necessary to connect large vessels and continuum nodes of the surrounding tissue.

The input parameters used for the finite element model include a physiological inlet blood pressure of 20 mmHg and an outlet pressure of 10 mmHg, simulating the typical pressure gradient across capillaries. The concentration of the solute at the inlet is set to 20 molars, with a zero-molar concentration at the outlet, enabling a strong driving force for mass transport. Blood viscosity is assumed to be equivalent to that of water, with a value of 1 × 10^−3^ Pa·s. Convective transport through the capillary wall is modeled using a leakage coefficient of 1 × 10^−11^, while diffusive transport is represented by a capillary wall permeability of 100. Diffusion within the capillaries is characterized by a high coefficient of 1 × 10^4^ mm^2^/s, reflecting rapid intravascular transport, whereas in the brain tissue, diffusion is significantly slower, with a coefficient of 0.2 mm^2^/s. The flow through the brain parenchyma is further governed by a Darcy coefficient of 1 × 10^−13^ mm^2^/s, indicating extremely low permeability and slow interstitial fluid movement.

The pre-processing and model generation stages are performed using the CAD FiS (Fields and Solids) software. Model construction begins with the selection of an appropriate computational module within the CAD environment, followed by the specification of geometrical configurations, material constitutive models, temporal discretization parameters, and other relevant simulation settings. The user is guided through specialized dialog interfaces to define the geometric properties of discrete solid domains—including mesh resolution and domain boundaries—as well as fluid flow characteristics, associated boundary conditions, mesh generation criteria, and solid material parameters.

The resulting finite element (FE) model data are exported in a proprietary *.dat format, which serves as the primary input for the PAK FE solver. Upon completion of the numerical simulation, output data—comprising fields such as displacements, velocity vectors and scalars, and pressure distributions—are written into a *.unv file. This file is automatically processed by the CAD FiS postprocessor module for visualization. Alternatively, simulation results can be exported in a *.vtk format, compatible with the ParaView platform for advanced post-processing and visualization.

The output files (*.unv or *.vtk) encapsulate nodal-level data for multiple physical fields, including displacements, shear stress distributions, and nodal velocity vectors. CAD FiS supports a comprehensive suite of visualization techniques, enabling scalar and vector field representation, point-based visualizations, and sectional analysis Via cutting planes.

Boundary conditions are prescribed at the inlet of capillaries (20 [M]) and outlet of veins (0 [M]) (denoted in Figure 6). Material parameters were taken from the literature to correspond to the mouse brain environment (such as [83]), with the addition of different diffusion (values were estimated using the Stokes–Einstein equation, Equation (1)) and clearance coefficients (values were estimated using the ADMET analysis) [84] for three different compounds (given in Table 4). The simulation lasts for 400 s, divided into 40 equal-time steps.

**Figure 7:**
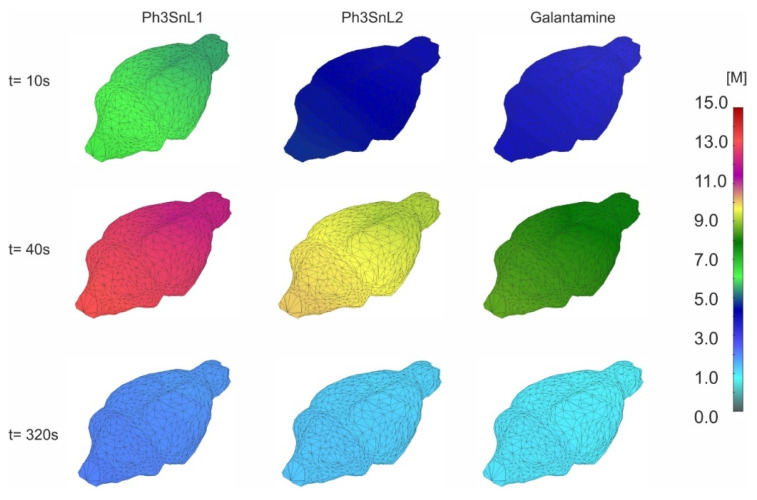
Mean concentration evolution inside the capillary and tissue domain within the mouse brain model, representing all the compounds, for t = 10 s, t = 40 s and t = 320 s.

The concentration field for three different time steps, in the vertical plane, for the surrounding tissue, and all the compounds from Table 4, is shown in Figure 7. Notably, the concentration reaches maximum values for all the compounds at 40 s, where differences in concentration values and clearance coefficient come from the diffusion coefficient variance. At the end of the diffusion process (Figure 6, t = 320 s), the concentration of the compounds drops to zero, due to the inlet concentration bolus profile.

## 4. Conclusions

Based on a comprehensive analysis of both experimental and computational data, it can be concluded that triphenyltin(IV) compounds, particularly when immobilized into mesoporous silica **SBA-15~Cl**, exhibit notable neuroprotective potential. Among the tested complexes, **SBA-15~Cl|Ph_3_SnL_2_** demonstrated the strongest acetylcholinesterase (AChE)-inhibitory activity, with a corrected IC_50_ value of 0.58 µM—approximately 26 times more potent than the reference drug galantamine (IC_50_ = 15.00 µM). This correction was made based on the actual content of the active complex, excluding the contribution of the SBA-15 carrier. These findings were further supported by molecular docking studies, where **Ph_3_SnL_2_** exhibited the lowest binding free energy toward **AChE** (ΔG_bind_ = −10.31 kcal/mol) and the lowest inhibition constant (K_i_ = 0.027 µM), outperforming galantamine (ΔG_bind_ = −8.85 kcal/mol; K_i_ = 0.33 µM).

The stability and strength of the AChE–ligand complexes were further confirmed by molecular dynamics simulations and MM/GBSA binding free energy calculations, which revealed thermodynamically favorable interactions: −29.53 kcal/mol for **AChE–Ph_3_SnL_1_** and −32.55 kcal/mol for **AChE–Ph_3_SnL_2_**. These results underscore the high binding affinity of the compounds and the relevance of hydrophobic interactions in selective enzyme inhibition.

Additionally, transport simulations using a numerical mouse brain model—featuring an immersed network of large blood vessels and realistic diffusion parameters—demonstrated efficient diffusion and distribution of the compounds throughout the surrounding brain tissue. Peak concentrations were reached during the early phase of the simulation (t = 40 s), followed by a gradual decline in accordance with the bolus inlet profile. These results confirm the compounds’ ability to penetrate brain tissue and highlight the influence of molecular diffusivity and clearance rates on their retention within the central nervous system.

In conclusion, immobilization of triphenyltin(IV) compounds onto SBA-15 nanostructures not only enhances their bioactivity but also improves their distribution profile within the CNS—both of which are critical factors for the development of new neuroprotective agents. Future studies should focus on evaluating their toxicity, pharmacokinetics, and antioxidant properties to fully validate their therapeutic potential for treating neurodegenerative disorders.

## Figures and Tables

**Table 1:** Inhibitory potential of functionalized organotin(IV) compounds and galantamine against acetylcholinesterase (**AChE**), expressed as apparent and corrected IC_50_ values (µmol/L).

Compounds	SBA-15~Cl|Ph_3_SnL_1_	Ph_3_SnL_1_	SBA-15~Cl|Ph_3_SnL_2_	Ph_3_SnL_2_	Galantamine
IC_50_ (µM) Apparent	23.93 ± 0.51	105.81 ± 0.58	13.44 ± 0.28	88.00 ± 0.51	15.00 ± 0.46
IC_50_ (µM) Corrected	1.07	–	0.58	–	–

**Table 2:** Important thermodynamic parameters (kcal mol^−1^) for the most stable conformation of investigated compounds in the active site of acetylcholinesterase (**AChE**) determined after molecular docking simulation: inhibition constants (*K*_i_, µM) and binding free energy (Δ*G*_bind_, kcal mol^−1^) values obtained from various energy components such as total internal energy (Δ*G*_total_), torsional free energy (Δ*G*_tor_), unbound system’s energy (Δ*G*_unb_), electrostatic energy (Δ*G*_elec_), and the sum of dispersion and repulsion (Δ*G*_vdw_), hydrogen bond (Δ*G*_hbond_), and desolvation (Δ*G*_desolv_).

Complexes	ΔG*_bind_*	K_i_ (µM)	ΔG*_inter_*	ΔG*_vdw+hbond+desolv_*	ΔG*_elec_*	ΔG*_total_*	ΔG*_tor_*	ΔG*_unb_*
**AChE**								
**Ph_3_SnL_1_**	−9.65	0.085	−11.57	−11.40	−0.17	−1.58	1.92	−1.58
**Ph_3_SnL_2_**	−10.31	0.027	−12.51	−12.35	−0.16	−1.69	2.20	−1.69
**Galantamine**	−8.85	0.330	−9.45	−9.05	−0.40	−0.77	0.60	−0.77
**Donepezil**	−11.27	0.0056	−13.06	−12.96	−0.10	−1.02	1.79	−1.2

**Table 3:** Binding energies throughout the simulation timeframe obtained through MM/GBSA and MM/PBSA methodologies.

Compounds	MM/GBSA	MM/PBSA
**AChE-Ph_3_SnL_1_**	−29.53	7.82
**AChE-Ph_3_SnL_2_**	−32.55	1.84

**Table 4:** Diffusion and clearance coefficients of organotin(IV) compounds (**Ph_3_SnL_1_** and **Ph_3_SnL_2_**) compared to the reference drug galantamine.

Compound Abbreviations (mL s^−1^ kg^−1^)	Diffusion Coefficient (mm^2^s^−1^)	Clearance Coefficient
**Ph_3_SnL_1_**	4.31 × 10^−4^	9.356
**Ph_3_SnL_2_**	4.24 × 10^−4^	8.015
**Galantamine**	5.17 × 10^−4^	6.176

## Data Availability

Data are contained within the article and Supplementary Materials.

## Supplementary Materials

The following supporting information can be downloaded at: https://www.mdpi.com/article/10.3390/nano15120914/s1; Figure S1: 3D (a,b) and 2D (c,d) representations of the inhibitors, galantamine and donepezil, within the active site of the AChE enzyme are presented. The red α-helices correspond to the helical regions of the AChE enzyme, which play a crucial role in maintaining the protein’s structural integrity and overall stability. The green loops and β-turns represent flexible regions of the enzyme, essential for facilitating conformational changes required for effective ligand binding. Numerical values, expressed in angstroms (Å), denote the interatomic distances, while different colors in the representations correspond to various types of intermolecular interactions, as detailed in the accompanying legend.
