# Supplementary material for: (Alkyl-ω-ol)triphenyltin(IV)-Loaded Mesoporous Silica as Biocompatible Potential Neuroprotectors: Evaluation of Inhibitory Activity Against Enzymes Associated with the Pathophysiology of Alzheimer’s Disease
Source: Nanomaterials (Basel). 2025 Jun 12;15(12):914. doi: 10.3390/nano15120914 (PMC12195829; doi:10.3390/nano15120914)
Supplement: Supplementary file 1 [file nanomaterials-15-00914-s001.zip › nanomaterials-3632245-supplementary.pdf]

Supplementary material

for

(Alkyl-ω-ol)triphenyltin(IV)-Loaded Mesoporous Silica as Biocompatible Potential Neuroprotectors: Evaluation of Inhibitory Activity Against Enzymes Associated with the Pathophysiology of Alzheimer's Disease

Kristina Milisavljević [1,2], Žiko Milanović [1,*], Jovana Matić [1,3], Marko Antonijević [1] , Vladimir Simić [1], Miljan Milošević [1,4], Marijana Kosanić [3] and Goran N. Kaluđerović [5,*]

[1] University of Kragujevac, Institute for Information Technologies, Jovana Cvijića bb, 34000 Kragujevac, Serbia;

[2]University of Kragujevac, Faculty of Science, Department of Chemistry, Radoja Domanovića 12, 34000 Kragujevac, Serbia;

[3] University of Kragujevac, Faculty of Science, Department of Biology, Radoja Domanovića 12, 34000 Kragujevac, Serbia;

[4] Belgrade Metropolitan University, Tadeuša Koćuška 63, 11000 Belgrade, Serbia;

[5] University of Applied Sciences Merseburg, Department of Engineering and Natural Sciences, Eberhard-Leibnitz-Straße 2, 06217 Merseburg, Germany;

***Corresponding author's e-mail address:** ziko.milanovic@uni.kg.ac.rs, goran.kaluderovic@hs-merseburg.de

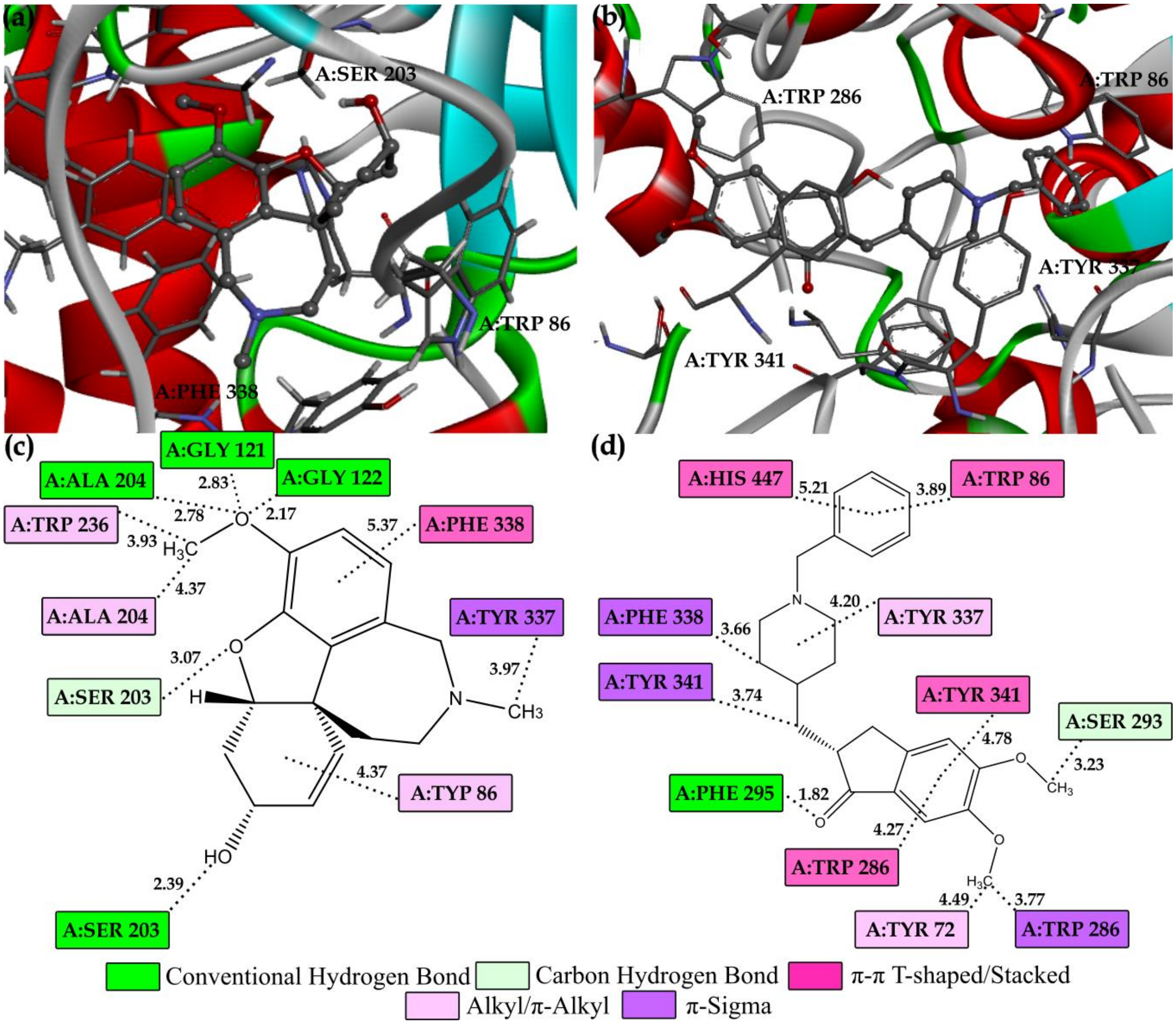

Figure S1. 3D (a, b) and 2D (c, d) representations of the inhibitors, **galantamine** and **donepezil**, within the active site of the **AChE** enzyme, are presented. The red α-helices correspond to the helical regions of the **AChE** enzyme, which play a crucial role in maintaining the protein's structural integrity and overall stability. The green loops and β-turns represent flexible regions of the enzyme, essential for facilitating conformational changes required for effective ligand binding. Numerical values, expressed in angstroms (Å), denote the interatomic distances, while different colors in the representations correspond to various types of intermolecular interactions, as detailed in the accompanying legend.
